# IJMS Special Issue—Molecular Mechanisms of Diabetic Kidney Disease 2.0

**DOI:** 10.3390/ijms26157315

**Published:** 2025-07-29

**Authors:** Ligia Petrica

**Affiliations:** 1 Department of Internal Medicine II, Division of Nephrology, “Victor Babes” University of Medicine and Pharmacy, No. 2, Eftimie Murgu Sq., 300041 Timisoara, Romania; petrica.ligia@umft.ro; 2 Centre for Molecular Research in Nephrology and Vascular Disease, Faculty of Medicine, “Victor Babes” University of Medicine and Pharmacy, No. 2, Eftimie Murgu Sq., 300041 Timisoara, Romania; 3 Centre for Cognitive Research in Neuropsychiatric Pathology (Neuropsy-Cog), Faculty of Medicine, “Victor Babes” University of Medicine and Pharmacy, No. 2, Eftimie Murgu Sq., 300041 Timisoara, Romania; 4 Center for Translational Research and Systems Medicine, Faculty of Medicine, “Victor Babes” University of Medicine and Pharmacy, No. 2, Eftimie Murgu Sq., 300041 Timisoara, Romania; 5 County Emergency Hospital Timisoara, 300723 Timisoara, Romania

Diabetic kidney disease (DKD) is a major cause of chronic renal involvement in both type 1 and type 2 diabetes mellitus (DM) and may be ascribed to a high percentage of patients referred to renal replacement therapies worldwide [1].

In their paper, Mazzier et al. [2] present an in-depth overview with regard to the main mechanisms involved in the pathogenesis and progression of DKD. They analyze the metabolic factors, oxidative stress, inflammatory pathways, fibrotic signaling, and hemodynamic mechanisms. This review summarizes the therapeutic options linked to specific molecular mechanisms of DKD, including renin–angiotensin–aldosterone system blockers, SGLT2 inhibitors, mineralocorticoid receptor antagonists, glucagon-like peptide-1 receptor agonists, endothelin receptor antagonists, and aldosterone synthase inhibitors. The therapeutic approach to DKD emphasizes the importance of all new classes of drugs in the clinical practice, which establish the main pillars of a personalized therapy for patients with DKD.

Autophagy and mitophagy are critical cellular processes that maintain homeostasis by removing damaged organelles and promoting cellular survival under stress conditions. In the context of diabetic kidney disease, these mechanisms play essential roles in mitigating cellular damage. The review by Stanigut et al. [3] provides an in-depth analysis of the recent literature on the relationship between autophagy, mitophagy, and diabetic kidney disease, highlighting the current state of knowledge, existing research gaps, and potential areas for future investigations.

The roles of the PINK1/Parkin pathway of mitophagy and the mitophagy receptor pathway and the key theories on autophagy and mitophagy in kidney disease are presented.

The authors focus on instruments and methods for assessing autophagy and mitophagy in kidney disease. The relation between autophagy and chronic kidney disease is discussed bearing in mind the role of autophagy in DKD. A special focus is on the AMPK-mTOR-Sirt1 pathway, the AMPK pathway, and the Sirt1 pathway. The main part of the review deals with the role of mitophagy in DKD. The authors correctly address the current gaps in knowledge, such as the heterogeneity in the autophagy response. Moreover, the mechanisms of regulation of mitophagy in DKD are presented. Also, limitations in current research are analyzed, especially limited human studies and context-dependent effects of autophagy.

The authors introduce future research directions, such as targeted therapies for autophagy and mitophagy modulation, in order to translate experimental and clinical data from preclinical to clinical trials and discover new therapeutic agents.

The review by Park K. et al. [4] encompasses an extensive presentation and analysis of the susceptibility of T1DM, while also reporting particularities to the Asian population, more specifically to Korean patients.

Type 1 diabetes mellitus (T1DM) is generally viewed as an etiologic subtype of diabetes mellitus caused by the autoimmune destruction of the insulin-secreting β-cells. It is now accepted that this type of DM can be present at any age. Etiologic heterogeneity that involves a varying incidence of a non-autoimmune subgroup of T1DM related to insulin deficiency has been proposed, a phenomenon which is associated with a decreased β cell mass.

To date, the immunologic pathogenesis of T1DM focusses on the permissible role of β-cells. There are crucial anti-islet autoantibody assays, such as the following: insulin, GAD65, IA-2, Zinc Transporter 8, Chromogranin A, and IGRP.

Although the diagnosis of T1DM rests on the detection of humoral antibodies directed against β-cell antigens, newly developed assays show that β-cell autoimmunity is primarily a T-cell-mediated process. CD4+ and CD8+ T-cells may be also implicated in the pathogenesis of T1DM. Experimental studies in transgenic mice reveal that CD8+ T-cells alone are capable of β-cell destruction. However, CD4+ islet-specific T-cell clones from the NOD mouse are also capable of inducing diabetes after adoptive transfer.

These results suggest that islet cell autoimmunity is also mainly genetically determined. It has now become evident that there are both susceptible and protective alleles at DRB1, DQA1, and DQB1 loci.

Several genome-wide association studies have revealed other genes outside the HLS system associated with T1D. Other genes that might influence the T1D risk include IL2RA (encoding α subunit of the IL-2 receptor), interleukin genes (mainly IL-4 and IL-13), PTPN2 (protein tyrosine phosphatase, non-receptor type 2), IFIH1 (interferon-induced helicase), BACH2 (basic leucine zipper transcription factor 2), GLIS3 (Gli-similar 3 protein), and ubiquitin-associated SH3.

The authors discuss in detail the fact that T1DM can be accepted also as an Adult-Onset Type 1 Diabetes. Moreover, they provide data about the concept of T1DM as an ambiguous autoimmune disease and a β-cell disease.

At the end of the review, the authors emphasize the role of diet and microbiota dysbiosis of the gastrointestinal tract elicited by changes in intestinal microbiota. An increased Bacteroidetes-to-Firmicutes ratio has been correlated with the expression of anti-islet autoantibodies and the onset of T1DM.

The authors conclude that different genetic susceptibilities or different genetic and environmental interactions might be involved in the etiology of T1DM and autoimmunity.

In their study, Dorflinger G.H. et al. [5] report that chronic inflammation plays an important role in the development of DN through the binding of MBL to hyperglycemia-exposed renal cells.

The BTBR OB mouse model of type 2 diabetes was used to investigate the role of the complement factor mannan-binding lectin (MBL) in diabetic nephropathy. This is an animal model that exhibits obesity and hyperphagia, develops profound insulin resistance and hyperinsulinemia, and progresses to diabetes. As the model exhibits hypotension, the factors related to hypertension in the pathophysiology of nephropathy are bypassed.

Renal cryosections from OB mice showed increased MBL-C and C4 deposition in the glomeruli and increased macrophage infiltration. Isolated glomeruli revealed significantly higher MBL protein levels compared to the OB and WT mice, and no renal MBL expression was detected.

The complement system is a central part of the innate immune system and plays a vital role in the first line of defense. The present results support our hypothesis that the autoreactivity of MBL towards kidney tissue is increased in a mouse model of type 2 diabetes and nephropathy. Furthermore, the authors showed the activation of the complement system by measuring MBL and C4b staining of glomeruli, but no signs of local transcription of MBL in the kidney cortex were observed. This supports the theory that prolonged hyperglycemia in diabetes can lead to glycoprotein alterations, which may enable adverse complement activation through the binding of MBL to neoepitopes.

The results of the study in the OB mouse model of T2D and nephropathy show that inflammation, initiated via the recognition of altered self-cells by MBL, plays a crucial role in the development of DKD.

Due to a mutation in the leptin gene, the OB mice are heavily obese as compared to the WT BTBR mice, and fat was visually accumulated in all organs. The renal damage, which was confirmed by morphology, the UACR, and cystatin C levels, was not found to be caused by kidney fibrosis. The study demonstrates increased levels of deposition of fibronectin in the glomeruli of the OB mice, but no difference was found in collagen deposition. A significant decrease was observed in the gene expression of collagen, the matrix metalloproteinase MMP-2, TIMP-2, and fibronectin in the kidney tissue, and no difference was observed in the expression of CTGF, MMP-9, TIMP1, or TGF-β.

This supports the idea that the renal damage in this model is primarily a result of inflammation, which may progress to renal fibrosis. In support of the importance of inflammation, we found highly increased C3 mRNA and circulating C3 levels in the OB mice, which is likely to be regulated by increased TNF-α mRNA in the OB mice.

Serine proteinase inhibitors (serpins) are intracellular proteins, while most of their identified targets are extracellular. They are distinguished by their unique mechanism of action, in which they irreversibly inhibit their target protease by undergoing a significant conformational change that disrupts the target’s active site. Serpins regulate a variety of biological processes, such as coagulation and inflammation, through protease inhibition [5]. Despite the numerous genetic loci that have been associated with the disease in T2DM, the genetic architecture of DKD remains unclear until today. In contrast to SERPINE1, the contribution of SERPINB2 has not been examined in DKD.

Tziastoudi M. et al. [6] conducted the first genetic association study of SERPINB2 to elucidate its role in DKD. In this study, to elucidate the contribution of the SERPINB2 gene to the pathogenesis of diabetic kidney disease in the context of type 2 diabetes mellitus (T2DM), four tag single-nucleotide polymorphisms (SNPs) were selected for genotyping in a case–control study of Caucasians.

This study examined whether specific variants in the SERPINB2 gene, encompassing four tag SNPs, are linked to the progression of type 2 diabetes mellitus and the development of diabetic kidney disease in the context of this type of diabetes mellitus. The analysis did not reveal any significant association between SERPINB2 variants and DKD, indicating no implication of SERPINB2 variants in the risk or development of the disease.

The lack of a significant association between SERPINB2 variants and DKD may indicate that these genetic variants do not have a major role in the progression of T2DM and in the risk or development of DKD. This could be because SERPINB2 is involved in fibrinolysis, a pathway that may not be central to the processes that cause diabetic kidney damage. SERPINB2 variants may not have a large enough effect on their own to contribute significantly to DKD risk, especially in the presence of stronger genetic and environmental factors. The present association study regarding SERPINB2 SNPs (rs4941230, rs3819335, rs13381217, rs6140) did not reveal any significant association between SERPINB2 variants and DKD.

In their previous studies, Balogh D.B. et al. [7] demonstrated that activating the Sigma-1 receptor (S1R) with fluvoxamine (FLU) protects against acute kidney injury by inhibiting inflammation and ameliorating the effect of hypoxia. Based on these observations, in their current study, the authors hypothesized that FLU might exert a similar protective effect in DKD. The effects of FLU on inflammation, hypoxia, and fibrosis were tested in human proximal tubular cells and normal rat kidney fibroblasts.

The Sigma-1 receptor (S1R) is a highly conserved chaperone protein primarily studied in the central nervous system, but it is also expressed in peripheral tissues [8]. S1R modulates several cellular processes, including calcium signaling, inflammation, oxidative stress, and apoptosis.

The in vitro experiments carried out in rats focused on modulating the S1R, a highly conserved chaperone protein primarily studied in the central nervous system regulating key cellular processes, including inflammation and hypoxia. As an inter-organelle signaling molecule, S1R is also present in peripheral tissues. In particular, the research group of the authors has identified the specific renal localization of this receptor, highlighting its potential in different kidney disease. Their promising preclinical results suggest that S1R activation provides renoprotective effects in acute models.

In their study, the authors assessed the effect of S1R agonist FLU through in vivo and in vitro models, particularly focusing on the critical pathological pathways.

The study showed elevated levels of KIM-1 and NGAL in diabetic rats, and FLU treatment reduced urinary KIM-1 and NGAL levels and the renal Havcr1 (KIM-1) expression, further supporting its protective role in maintaining tubular integrity. Long-term FLU treatment improved renal function and reduced serum creatinine and BUN levels in the STZ-induced T1DM rats, demonstrating its ability to protect renal function.

In the study performed by Balogh et al., diabetic rats exhibited excessive glomerular basement membrane thickening and mesangial matrix expansion. FLU treatment attenuated these alterations, suggesting that S1R activation not only preserves kidney function but also plays a crucial role in preventing structural damage. To further investigate LPS-induced NF-κB activation, the mRNA expression of inflammatory mediators was also measured. LPS treatment markedly increased NFKB, IL1B, IL6, and TNF, while FLU significantly halted NF-κB downstream signaling and reduced hypoxia-induced TGFB1 expression, suggesting that its antifibrotic effects may be partly mediated by modulating the hypoxic response.

The in vitro findings revealed that FLU suppressed TGF-β1-induced fibroblast transformation and ECM deposition. By inhibiting the expression of Col1a1, Col3a1, and Fn, FLU demonstrated its ability to reduce fibrosis at the cellular level.

These findings suggest that FLU effectively modulates fibrotic pathways across different cell types and tissues, highlighting its potential as a therapeutic strategy for mitigating renal fibrosis.

FLU improved renal function and reduced glomerular damage and tubulointerstitial fibrosis. It also mitigated inflammation by reducing TLR4, IL6, and NFKB1 expressions and moderated the cellular response to tubular hypoxia. Additionally, FLU suppressed TGF-β1-induced fibrotic processes and fibroblast transformation. These findings suggest that S1R activation can slow down DKD progression and protect renal function by modulating critical inflammatory, hypoxic, and fibrotic pathways; therefore, it might serve as a promising novel drug target for preventing DKD.
